# Challenges in Clinicogenetic Correlations: One Gene – Many Phenotypes

**DOI:** 10.1002/mdc3.13165

**Published:** 2021-03-02

**Authors:** Francesca Magrinelli, Bettina Balint, Kailash P. Bhatia

**Affiliations:** ^1^ Department of Clinical and Movement Neurosciences, UCL Queen Square Institute of Neurology University College London London United Kingdom; ^2^ Department of Neurosciences, Biomedicine and Movement Sciences University of Verona Verona Italy; ^3^ Department of Neurology University Hospital Heidelberg Heidelberg Germany

**Keywords:** clinicogenetic correlation, genotype, movement disorders, phenotype, phenotypic heterogeneity

## Abstract

**Background:**

Progress in genetics – particularly the advent of next‐generation sequencing (NGS) – has enabled an unparalleled gene discovery and revealed unmatched complexity of genotype–phenotype correlations in movement disorders. Among other things, it has emerged that mutations in one and the same gene can cause multiple, often markedly different phenotypes. Consequently, movement disorder specialists have increasingly experienced challenges in clinicogenetic correlations.

**Objectives:**

To deconstruct biological phenomena and mechanistic bases of phenotypic heterogeneity in monogenic movement disorders and neurodegenerative diseases. To discuss the evolving role of movement disorder specialists in reshaping disease phenotypes in the NGS era.

**Methods:**

This scoping review details phenomena contributing to phenotypic heterogeneity and their underlying mechanisms.

**Results:**

Three phenomena contribute to phenotypic heterogeneity, namely incomplete penetrance, variable expressivity and pleiotropy. Their underlying mechanisms, which are often shared across phenomena and non‐mutually exclusive, are not fully elucidated. They involve genetic factors (ie, different mutation types, dynamic mutations, somatic mosaicism, intragenic intra‐ and inter‐allelic interactions, modifiers and epistatic genes, mitochondrial heteroplasmy), epigenetic factors (ie, genomic imprinting, X‐chromosome inactivation, modulation of genetic and chromosomal defects), and environmental factors.

**Conclusion:**

Movement disorders is unique in its reliance on clinical judgment to accurately define disease phenotypes. This has been reaffirmed by the NGS revolution, which provides ever‐growing sequencing data and fuels challenges in variant pathogenicity assertions for such clinically heterogeneous disorders. Deep phenotyping, with characterization and continual updating of “core” phenotypes, and comprehension of determinants of genotype–phenotype complex relationships are crucial for clinicogenetic correlations and have implications for the diagnosis, treatment and counseling.

“Phenotype” is the observable or quantifiable characteristics of an individual – including findings of nongenetic investigations – which result from the interaction of its gene makeup with environmental factors. However, in a narrower sense, geneticists refer to phenotype as the set of specific features arising from the expression of one or few genes. In keeping with this, “genotype” is the genetic constitution of an individual, overall or at a specific locus, that is responsible of a given phenotype.^1^

The identification of the first disease genes in the early 1980s suggested simplistically that phenotypes could be precisely predicted if genotypes were determined, therefore enabling consistent genotype–phenotype correlations.^2^ However, advances in genetics – particularly the advent of next‐generation sequencing (NGS) – have revealed that the relationship between genotype and phenotype is not straightforward, even for single‐gene disorders.^1^, ^2^ This has become more evident with the use of hypothesis‐free whole‐exome (WES) and whole‐genome sequencing (WGS) instead of candidate gene approaches to identify new disease genes.^2^, ^3^

Movement disorders (MD) is relatively unique among neurology subspecialities in its reliance on clinical assessment as well as clinicians' expertise and experience to accurately define disease phenotypes.^4^ Indeed, MD are highly heterogeneous conditions, often present as complex clinical pictures with overlapping manifestations, and almost invariably lack of diagnostic biomarkers.^4^ Equally, progress in genetics has enabled an unparalleled gene discovery and revealed unmatched complexity of genotype–phenotype associations in the field of MD.^5^ Consequently, MD specialists have increasingly faced challenges in clinicogenetic correlations, with implications for diagnosis, treatment and genetic counseling.

Mutations in different genes may account for the same “core” phenotype (genetic heterogeneity). For instance, variants in *NKX2‐1*, *ADCY5* and *PDE10A* can all manifest with early‐onset chorea,^6^ mutations in *PRRT2*, *MR‐1*, *SCN8A* and *SLC16A2* with paroxysmal kinesigenic dyskinesia,^7^ and defects in *GLRA1*, *GLRB* and *SLC6A5* with hyperekplexia.^8^ On the other hand, mutations in one and the same gene can cause multiple, often markedly different phenotypes (phenotypic heterogeneity), which is the topic of this article.

Three main phenomena contribute to phenotypic heterogeneity of monogenic disorders (Fig. 1). First, some individuals carrying a disease‐causing mutation in a gene may not express the corresponding disease phenotype, which is defined “incomplete penetrance”.^1^, ^9^, ^10^ Second, if the mutation is expressed, the disease phenotype can present with varying degrees of severity across carriers, therefore showing “variable expressivity”.^1^, ^10^ Finally, a mutation in a gene controlling two or more phenotypic traits may account for multiple, apparently unrelated, disease phenotypes as a result of “pleiotropy”.^1^, ^11^ Although being distinct concepts, penetrance, expressivity and pleiotropy are inter‐related and often underpinned by shared biomechanisms involving genetic, epigenetic and environmental factors. Stochastic (ie, random) events, which can occur at either the DNA, RNA, or protein level, might also play a role in phenotypic heterogeneity, but their relative contribution is hardly quantifiable (Fig. 2).^12^

**FIG. 1:**
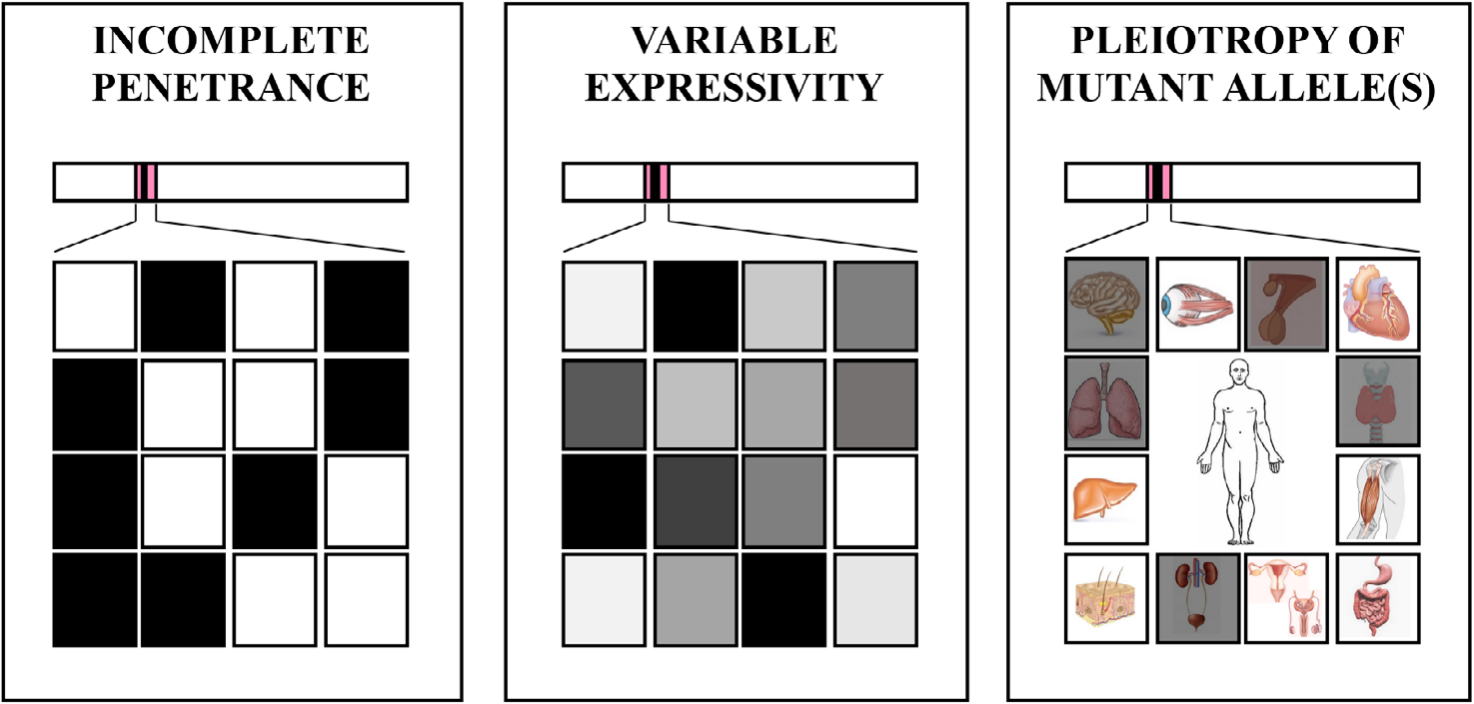
Phenomena contributing to phenotypic heterogeneity in monogenic disorders. *Left and middle boxes*. Squares represent individuals carrying the same variant in a gene. *Left box*. Shaded square means the individual manifests the disease phenotype. Non‐shaded square means the individual does not manifest the disease phenotype (non‐penetrance). *Middle box*. Shaded square means the individual manifests the disease phenotype with different degree of severity. Non‐penetrance (non‐shaded squares) can be viewed as an extreme endpoint of variable expressivity. *Right box*. Individual carrying a variant in a pleiotropic gene with multisystemic effects. In the example, a variant in the *NKX2‐1* gene encoding the thyroid transcription factor 1, with involvement (shaded squares) of the nervous system (chorea, choreoathetosis), pituitary gland (cystic mass), thyroid (congenital hypothyroidism), lung (neonatal respiratory distress, chronic interstitial lung diseases), and urinary system (megabladder).

**FIG. 2:**
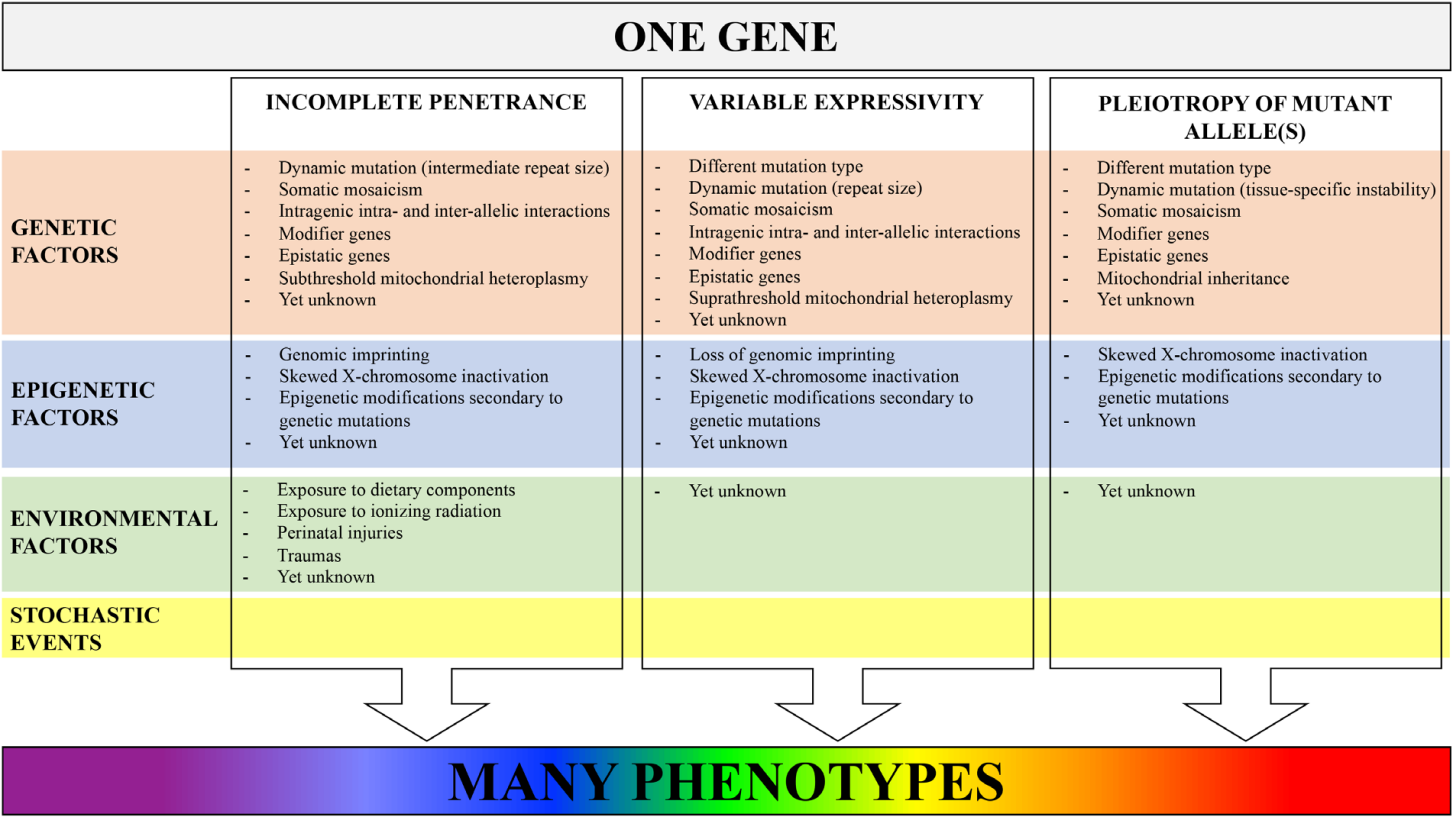
Overview of genetic and nongenetic (epigenetic, environmental) factors underpinning incomplete penetrance, variable expressivity and pleiotropy of mutant allele(s). Stochastic (ie, random) events might act at all levels and further contribute to phenotypic heterogeneity.

This review deconstructs the “One Gene – Many Phenotypes” paradigm providing examples of monogenic MD and neurodegenerative diseases. We analyze phenomena contributing to phenotypic heterogeneity and underlying genetic and nongenetic factors. We also discuss the evolving role of MD specialists in reshaping phenotypes in the NGS era.

## Phenomena Contributing to Phenotypic Heterogeneity

### Incomplete Penetrance

“Penetrance” of a monogenic disorder is the conditional probability that an individual carrying a mutation at the corresponding gene manifests the disease phenotype. If this probability does not equal 100% within a specific time period, the disorder displays “incomplete penetrance” (Fig. 1).^1^, ^9^, ^10^ We favor this expression over “reduced penetrance” since the latter gives more room to the currently evolving concept of nuances of expressivity rather than the all‐or‐none issue of the disease phenotype being manifested or not. Non‐penetrance can be regarded as an extreme endpoint of phenotypic heterogeneity.

Incomplete penetrance is most evident in autosomal dominant (AD) disorders, which, by definition, are manifested in heterozygotes and might therefore be expected to have full penetrance in carriers of a pathogenic variant.^1^ Hence, some AD disorders occasionally appear to “skip” a generation, meaning that individuals carrying an inherited or *de novo* mutation do not express the disease phenotype (asymptomatic carriers) but can transmit the mutant allele to the offspring.^1^ This occurrence, which is well‐described for example in *TOR1A*‐related dystonia (DYT‐*TOR1A*; OMIM# 128100),^13^, ^14^, ^15^ *SGCE*‐related myoclonus‐dystonia (OMIM# 159900),^16^, ^17^, ^18^ and many repeat expansion disorders,^19^ poses a challenge in defining the inheritance pattern, especially in small pedigrees.

Autosomal recessive (AR), X‐linked and mitochondrial disorders can also exhibit incomplete penetrance. In Wilson's disease (WD; OMIM# 277900), a discrepancy between the frequency of individuals carrying biallelic pathogenic variants in *ATP7B* (1:7000)^20^ and the prevalence determined by mass screening using ceruloplasmin in blood/urine (1–2:3000)^21^, ^22^ suggests the penetrance is not full, particularly for variants mapping outside a three‐exon hotspot gene region where 50% of WD‐causing mutations were identified in a UK population study.^23^ Penetrance is incomplete in females with premutation and mutation in *FMR1* causing X‐linked dominant fragile X‐associated tremor‐ataxia syndrome (FXTAS; OMIM# 300623) and fragile X syndrome (FXS; OMIM# 300624), respectively.^24^ Finally, disorders due to mutations in mitochondrial DNA (mtDNA) show incomplete penetrance^25^ either because the ratio between mutated and wild‐type mtDNA does not reach the “phenotypic threshold level” or despite the mutation being present in all mtDNA copies.^26^, ^27^

Among others, age, gender and ethnicity are drivers of incomplete penetrance in certain diseases.

#### Age‐Related Penetrance

Some monogenic disorders show late age of onset, with penetrance being very low in the first decades and increasing with age. Different mechanisms account for the slow development of adult‐onset neurogenetic disorders. For instance, mutant gene products or substrates of defective enzymes may accumulate slowly, and/or the pathogenesis involve a gradual process of neuronal loss requiring time before the number of surviving cells drops below a critical threshold or, at least theoretically, overcomes brain plasticity, finally causing symptom onset. This is exemplified by repeat expansion disorders,^19^ such as Huntington disease (HD; OMIM# 143100) and FXTAS, in which mutant alleles with “intermediate” number of repeats are often associated with incomplete penetrance and late presentation. Furthermore, the estimate prevalence of *LRRK2*‐related Parkinson's disease (PD; OMIM# 607060) among non‐Ashkenazi Jewish carriers of the monoallelic variant NM_198578.4:c.6055G>A (p.Gly2019Ser) was found to be 7.33% at 60 years, 29.17% at 70 years, and 42.52% at 80 years.^28^

#### Gender‐Related Penetrance

The effect of gender on penetrance has been observed in some single‐gene disorders. For instance, *GCH1*‐associated dopa‐responsive dystonia (OMIM# 128230) shows a 2.3 times higher mutation prevalence in females than males.^29^ Mechanisms underlying female predominance are not understood, although sexual differentiation of mesencephalic dopaminergic neurons and sex hormones might be contributors to gender‐related vulnerability to tetrahydrobiopterin deficiency.^29^

#### Ethnicity‐Related Penetrance

Penetrance may be influenced by ethnicity, as demonstrated in *LRRK2*‐PD, in which age‐related penetrance of the p.Gly2019Ser variant was found to significantly differ between Tunisian Arab‐Berbers and Norwegians, the former having a median age at onset 10 years earlier than the latter.^30^ Moreover, in *ATXN3*‐related spinocerebellar ataxia (formerly SCA3; OMIM# 109150) Asians have a mean age of onset 4.75 years and 6.64 years higher than Caucasians and African Americans, respectively.^31^

In many cases determinants of incomplete penetrance are unknown. These might include the interaction among multiple factors, small or large effect size, etc., but any consideration in this regard is purely speculative.

### Variable Expressivity

“Expressivity” is the extent to which a given genotype is expressed at the phenotypic level. When the same genetic variant is expressed and shows quantitatively different effects among distinct individuals, even among members of the same family (intrafamilial variability), the corresponding disease phenotype displays “variable expressivity” (Fig. 1).^1^, ^10^ Variable expressivity is recognized in monogenic disorders with all patterns of inheritance and represents a major contributor to phenotypic heterogeneity.

Several underpinnings of variable expressivity have been proposed so far and partly overlap with those of incomplete penetrance. For instance, variable expressivity can correlate with the repeat size (ie, length of the expansion) in repeat expansion disorders^10^ or with different mutation types in the same gene, with missense, nonsense and frameshift variants resulting in partially functional or non‐functional gene products. Nuances of expressivity may also be related to tissue‐dependent thresholds of susceptibility to the same genetic defect; namely, tissues where a given mutation is actually expressed can differ in the level of protein deficiency at which symptoms manifest (with different degree of severity). Furthermore, in mitochondrial disorders, variable expressivity can depend on different suprathreshold degrees of heteroplasmy of mutant mtDNA. Finally, inter‐individual variable expressivity may reflect differences in the allelic constitution of the rest of the genome or the interplay between (epi)genetic and environmental factors.

Overall, variable expressivity stands among the most challenging hindrances in the interpretation of genetic variants. In many cases, such as in *ATP1A3‐* and *TUBB4*‐related disorders,^32^, ^33^ it remains controversial whether phenotypic heterogeneity is due to variable expressivity (ie, phenotypic spectrum of the same disease), pleiotropy (ie, discrete phenotypes related to the involvement of different organs or different neuronal subpopulations in exclusively neurological disorders), or both.^34^ Elucidating the molecular basis of variable expressivity, for example by implementing functional studies on the effect of different variants in the same gene, may be crucial in genotype–phenotype correlations.

### Pleiotropy of Mutant Allele(s)

“Pleiotropy” is the phenomenon whereby a single gene influences two or more distinct phenotypic traits.^1^, ^11^ Germline mutations in pleiotropic genes account for disease phenotypes showing selective involvement of a subset of tissues, organs or systems which constitutionally express those genes (Fig. 1). This occurs because all cells are structurally and functionally specialized through diverse gene transcriptional profiles despite containing an identical genome except for postzygotic (somatic) mutations^11^; namely, under physiological conditions, cells only express a fraction of genes required for their baseline (“housekeeping” genes) and specific (“luxury” genes) structure and functions. Notwithstanding, individuals carrying mutations in a pleiotropic gene may present with high‐level phenotypic heterogeneity due to the qualitatively (eg, different organs/systems affected) and quantitatively (ie, severity) wide spectrum of multisystemic involvement.

Pleiotropy has initially been described in monogenic disorders. In this case, mutations in genes that are expressed by different cell types cause a constellation of apparently unrelated clinical features secondary to multiorgan/multisystemic dysfunctions. However, pleiotropy has revealed itself as a more widespread phenomenon occurring also for small‐size effect variants. A review of genome‐wide association studies revealed that ~17% of genes and 4%–5% of genetic variants are pleiotropic, and this is often observed in neurological disorders.^11^ Despite its frequency and contribution to phenotypic heterogeneity, little is known about mechanisms underlying pleiotropy, as well as about properties of pleiotropic proteins. A pleiotropic mutation can account for a multisystemic disorder in various ways.^35^ Among others, the resulting gene product can be used for the same biochemical purpose in multiple biological pathways. The mutant protein can also have more than one function through different domains or interaction with different partners in different cell types. Moreover, the defective gene product may affect a singular molecular function, whose alteration secondarily impacts on other functions with a cascade mechanism.^35^ Analysis of multisystemic disorders in humans has shown that pleiotropy is more common in genes encoding essential proteins and “hub” proteins (proteins with multiple interactors),^11^ thus confirming the need to study interactome networks to further progress in our understanding of genetic diseases.^36^

Examples of genes encoding pleiotropic proteins are *VCP* and *NKX2‐1*.^37^, ^38^ *VCP* encodes the valosin‐containing protein, a ubiquitously expressed protein involved in several cellular activities, including cell cycle control, membrane fusion and the ubiquitin‐proteasome degradation pathway.^34^ Pathogenic variants in *VCP* account for a rare AD multisystemic disorder characterized by inclusion body myopathy, Paget disease of bone and frontotemporal dementia (OMIM# 605382).^37^ They have also been linked to amyotrophic lateral sclerosis (OMIM# 613954)^39^ and Charcot–Marie–Tooth disease type 2Y (OMIM# 616687).^40^ *NKX2‐1* codes for the thyroid transcription factor 1, a nuclear protein expressed during early development of the forebrain (especially basal ganglia and hypothalamus), lung, and thyroid.^35^ Heterozygous pathogenic variants in *NKX2‐1* have been linked to the brain‐lung‐thyroid syndrome (OMIM# 610978), a multiorgan disorder characterized by early‐onset chorea, respiratory distress syndrome, and congenital hypothyroidism.^38^ Dysfunctions of the pituitary gland and urinary system (pyelectasis and megabladder) can be associated with mutations in *NKX2‐1* (Fig. 1).^41^, ^42^

Pleiotropic genes represent a major challenge when analyzing and prioritizing genes and genetic variants for their potential association with disease phenotypes.

## Mechanisms underlying phenotypic heterogeneity

Genetic, epigenetic and environmental factors may influence penetrance, expressivity and pleiotropy of mutant alleles in a non‐mutually exclusive manner, even in monogenic disorders, whose phenotype, by definition, is largely determined by the genotypic status at just one locus (Fig. 2). In many cases, the aforementioned phenomena display overlapping boundaries at the molecular level.

### Genetic Factors

#### Different Mutation Types

Different mutation types in one and the same gene may either cause variable expressivity (ie, mild or severe forms of the same disease phenotype), or account for rather different disorders, thus representing a molecular explanation for pleiotropy.

A subset of genes is highly dosage sensitive, meaning that changes in a gene dose and consequently in the amount of gene product are critically relevant. Changes in gene copy number (copy number variations) may account for disease by modifying the amount of protein product beyond normal limits.^43^, ^44^ Furthermore, certain point mutations have the same effect by reducing (loss‐of‐function) or amplifying (gain‐of‐function) gene expression. To oversimplify, disease‐causing missense mutations may result in gene products that still retain their function to some extent (partially functional proteins). On the contrary, nonsense and frameshift mutations lead to transcripts carrying a premature termination codon which are often subject to nonsense‐mediated mRNA decay,^45^ thus resulting in loss‐of‐function alleles, or can rarely escape depletion and be translated to truncated proteins with potential gain‐of‐function and/or toxic effects. However, the impact of a variant also depends on its location within the protein (eg, inside/outside functional domains, in sites that are critical for its tertiary and quaternary structures). For instance, missense mutations involving the catalytic site of an enzyme can have highly detrimental consequences. Phenotypic heterogeneity depending on different mutation types is exemplified by enzyme deficiency disorders, in which there is often a good correlation between gene product levels and phenotype severity. For example, the phenotypic spectrum related to the X‐linked gene *HPRT1* reflects quite predictably the residual activity of the enzyme hypoxanthine guanine phosphoribosyltransferase 1, which is involved in the synthesis of purine nucleotides through the purine salvage pathway.^46^ Loss‐of‐function mutations lowering the normal enzymatic activity <60% result in asymptomatic hyperuricemia or gout (OMIM# 300323), whereas neurological features appear when HPRT1 activity drops below 8%. With HPRT1 activity ~1.5% individuals manifest Lesch–Nyhan syndrome (OMIM# 300322) but can still have normal intelligence. A decrease in HPRT activity to <1.4% results in full Lesch–Nyhan syndrome, including choreoathetosis, pyramidal signs, self‐injurious behavior, and intellectual disability.^1^

Another example is glucose transporter type 1 (Glut1) deficiency syndrome, which is caused by heterozygous or, less frequently, biallelic mutations in the *SLC2A1* gene and has a wide phenotypic spectrum.^7^, ^47^ The classic phenotype (OMIM# 606777) is characterized by early‐onset chronic encephalopathy with pharmacoresistant epilepsy, acquired microcephaly, spasticity and MD. Milder phenotypes may manifest during childhood or adulthood with epilepsy, cognitive/behavioral issues and MD (most frequently paroxysmal exercise‐induced dyskinesia, but also ataxia), either isolated or in various combinations.^47^ A correlation between the specific type of *SLC2A1* pathogenic variant and the clinical severity has been observed.^47^, ^48^ Missense variants are found predominantly in milder phenotypes, possibly reflecting the presence of a partially functional Glut1 in the brain.^47^, ^48^ On the contrary, splice site, nonsense and frameshift mutations as well as exon and complete gene deletions occur almost only in the classic severe phenotype.^47^, ^48^

Among gain‐of‐function mutations, multiplications in the α‐synuclein gene (*SNCA*) account for AD parkinsonism, autonomic dysfunction and dementia with a gene dosage effect.^49^, ^50^, ^51^, ^52^ The clinical phenotype related to whole‐gene duplication (ie, one extra copy of wild‐type *SNCA*) resembles idiopathic PD, whereas triplication and quadruplication (ie, two and three extra copies, respectively) cause increasingly severe phenotypes of early‐onset rapidly progressive PD with dysautonomia and cognitive impairment.^49^, ^50^, ^51^, ^52^

Different types of monoallelic mutations in *CACNA1A*, which encodes the voltage‐gated P/Q‐type calcium channel subunit alpha‐1A, are associated with a number of different phenotypes, including SCA6 (OMIM# 183086),^53^ episodic ataxia type 2 (EA2; OMIM# 108500),^54^ familial hemiplegic migraine type 1 (FHM1; OMIM# 141500)^55^ with or without progressive cerebellar ataxia, benign paroxysmal torticollis of the infancy,^56^ early infantile epileptic encephalopathy,^57^ and paroxysmal head tremor.^58^ Of the first three allelic disorders reported, SCA6 is a polyglutamine disorder caused by a 20‐to‐33 CAG triplet expansion in exon 47 of *CACNA1A*,^53^ whereas EA2 and FHM1 are due to *CACNA1A* loss‐of‐function mutations and gain‐of‐function missense mutations, respectively.^54^, ^55^ *CACNA1A*‐related disease phenotypes described more recently have revealed that there is a wide phenotypic overlap between hemiplegic migraine, diverse forms of cerebellar dysfunction and epilepsy, and that genotype–phenotype correlation might be not as strict as initially reported. For example, both loss‐of‐function and gain‐of‐function *CACNA1A* mutations cause severe developmental epileptic encephalopathies in the spectrum of Lennox–Gastaut syndrome and congenital ataxia.^57^

#### Dynamic Mutations

**FIG. 3:**
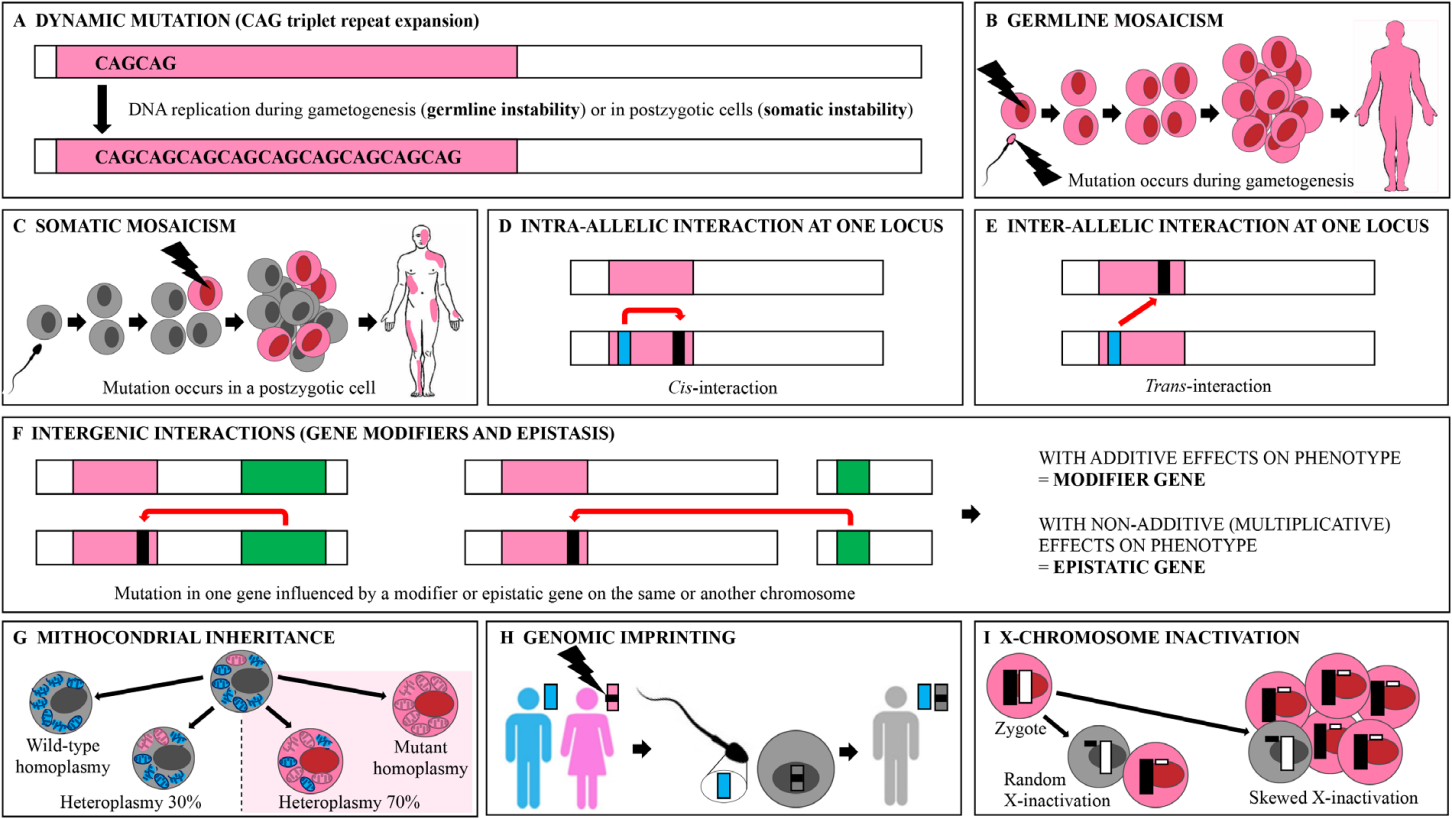
Schematic of genetic and epigenetic mechanisms underlying phenotypic heterogeneity. (**A**) Germline instability of a CAG triplet repeat expansion (dynamic mutation). (**B**) A *de novo* mutation (thunderbolt) occurring during the gametogenesis in a healthy individual is transmitted to the offspring (germline mosaicism). (**C**) Somatic mosaicism resulting from a *de novo* mutation (thunderbolt) in a postzygotic cell which is only carried by a fraction of somatic cells. (**D**) Modulation of gene expression resulting from intragenic intra‐allelic interaction (*cis*‐interaction). (**E**) Modulation of gene expression resulting from intragenic inter‐allelic interaction (*trans*‐interaction). (**F**) Modulation of gene expression by a modifier or epistatic gene which can map on the same (left) or another chromosome (right). (**G**) Mitochondrial inheritance deriving from random segregation of mitochondria during cell replication. The dashed line represents the “phenotypic threshold level” for the mutation of mitochondrial DNA to manifest (wild‐type mitochondria are blue, mutant mitochondria are pink). (**H**) Genomic imprinting through epigenetic mechanisms enables postzygotic cells to retain memory of the parental origin of an allele. In the example, a mutation in a gene maternally imprinted is not expressed in the offspring when transmitted by the mother. (**I**) X‐chromosome inactivation leads to functional inactivation of one copy of chromosome X in cells of female individuals to provide dosage compensation between the sexes. Skewed X‐inactivation occurs when the inactivation of one X‐chromosome is favored over the other (wild‐type allele is white, mutant allele is black).

Oligonucleotide repeat expansions are unstable (“dynamic”) mutations whose repeat size can change after DNA replication (Fig 3A).^59^ They account for more than 40 neurological disorders, including HD (CAG repeat expansion in *HTT*), FXTAS and FXS (CGG repeat expansion in *FMR1*), dentatorubral‐pallidoluysian atrophy (DRPLA; OMIM# 125370; CAG in *ATN1*), *C9orf72*‐related disorders (GGGGCC in *C9orf72*) and some SCA.^19^ However, the number of repeat expansion disorders is set to rise with the advent of long‐read sequencing and other technologies.^60^ Repeat instability can influence penetrance and expressivity, leading to intrafamilial intergenerational phenotypic heterogeneity. It may also occur within specific tissues and contribute to pleiotropy. Since repeat size correlates inversely with age of onset and phenotype severity in many repeat expansion disorders, repeat instability provides a molecular explanation for genetic anticipation.^19^

Repeat instability occurs during parent‐to‐offspring transmission (germline instability), with parent‐of‐origin biases possibly due to specific processes occurring during spermatogenesis or oocytogenesis. For example, paternal expansion bias is observed in HD and DRPLA, whereas paternal contraction and maternal expansion biases (modulated by epigenetics factors) are observed in FXTAS and FXS.^59^

Some repeat expansion disorders also exhibit tissue‐specific somatic repeat instability whose timing, pattern and tropism differ among disorders.^59^ For instance, it occurs throughout the lifetime for HD and DRPLA, while it occurs only in fetal tissues for FXS and FXTAS. The contribution of brain‐specific somatic repeat instability to the progression rate of neurodegenerative repeat expansion disorders is far from being clarified.^59^

#### Somatic Mosaicism

*De novo* pathogenic mutations can occur during gametogenesis, thus resulting in the presence of gametes with mutant alleles in otherwise healthy individuals (germline mosaicism, Fig 3B) and the transmission of the mutant alleles to the zygote.^61^ However, most mutations occur in cells at any time in postzygotic life due to endogenous errors in DNA replication and repair. As a result of postzygotic mutations, individuals are mosaic with genetically distinct cell populations characterized by different mutational load (somatic mosaicism, Fig 3C).^62^ Somatic mosaicism contributes to variable expressivity of single‐gene disorders through gene dose effect or by acting in addition with other genetic or epigenetic factors.^62^ It has been reported in individuals with no‐penetrance or mild presentation of Mendelian disorders, including AD *ADCY5*‐related early‐onset chorea (OMIM# 606703). Low‐level and tissue‐limited mosaicism represent major challenges for clinicogenetic diagnoses.^63^ NGS with deep sequence coverage and DNA extraction from tissues of interest enhance sensitivity and enable accurate quantification of the degree of somatic mosaicism.^61^, ^64^

#### Intragenic Intra‐ and Inter‐Allelic Interactions

Phenotypic heterogeneity may depend on intragenic interactions within the same allele (*cis*‐regulation, Fig 3D) or between the two alleles (*trans*‐regulation, Fig 3E). Prion diseases offer an example of intragenic intra‐allelic interaction.^65^ The missense mutation NM_000311.5:c.532G>A (p.Asp178Asn) in the prion protein gene (*PRNP*) results in two different phenotypes, either familial Creutzfeldt‐Jacob disease (fCJD; OMIM# 123400) or fatal familial insomnia (FFI; OMIM# 600072), depending on whether the *PRNP cis* codon 129 is methionine or valine, with the former typically associated with FFI and the latter with fCJD.^65^

An intragenic inter‐allelic interaction influences disease penetrance in DYT‐*TOR1A*.^13^ DYT‐*TOR1A* is almost invariably due to the heterozygous in‐frame deletion NM_000113.2:c.907_909delGAG (p.Glu303del) in the gene encoding the ATP‐binding protein torsinA.^13^ The penetrance of this variant is ~30%.^66^ The presence of the polymorphism NM_000113.2:c.646G>C (p.Asp216His) in *trans* configuration with the GAG deletion in *TOR1A* reduces the penetrance to ~3%.^14^

Furthermore, a significant intragenic inter‐allelic interaction between the non‐expanded (wild‐type) allele in *trans* and age at onset was observed in individuals with SCA1 (OMIM# 164400), SCA6 and SCA7 (OMIM# 164500). “Intermediate” wild‐type alleles interacting with the expanded allele decrease age at onset in SCA1 and SCA6, whereas short or medium wild‐type alleles interacting with the expanded allele decrease age at onset in SCA7.^67^

#### Intergenic Interactions (Modifier Genes and Epistasis)

Modifier and epistatic genes modulate the expression of a target disease gene by interacting from a distance with its locus with different levels of complexity (Fig 3F).^68^, ^69^, ^70^ Modifiers genes influence penetrance, expressivity, and pleiotropy of a mutant allele through simple, additive interactions with the target gene. By contrast, the crosstalk between the target disease gene and epistatic genes results in multiplicative (ie, non‐additive) effects which are more difficult to explore, such as masking the disease phenotype or expressing a new disease phenotype.

Different alleles at a modifier locus can have protective effects, resulting in late‐onset and/or milder disease phenotypes, or aggravating effects, by inducing young‐onset and/or severe phenotypes.^68^ For example, although individuals with HD carry the same type mutation (ie, CAG repeat expansion) in *HTT*, it is unlikely that two affected with exactly the same *HTT* CAG repeat size exhibit the same phenotype in terms of age of onset and clinical manifestations.^71^ The presence and size of an expanded CAG tract contributes only 60% on average to individual variation in HD age of onset, thus suggesting the presence of other (epi)genetic and environmental determinants. Age of onset in individuals with HD was found to be modulated by CAG repeat sizes in the normal range of *ATXN3*, *CACNA1A* and the androgen receptor gene.^71^

Another example comes from some SCAs, whose age at onset is influenced by other genes containing CAG repeats, namely *ATXN7* in SCA2 (OMIM# 183090), *ATXN2*, *ATN1* and *HTT* in SCA3, *ATXN1* and *ATXN3* in SCA6, and *ATXN3* and *TBP* in SCA7.^67^

Recently, in the *TOR1A*‐dystonia mouse model, reduced expression of torsinB encoded by the paralog *TOR1B* was found to cause a dose‐dependent worsening of twisting, whereas torsinB overexpression was proven to rescue torsinA deficiency.^72^ These findings identify torsinB as a potent modifier of torsinA loss‐of‐function phenotypes and suggest that enhancing neuronal torsinB expression in neurons at the appropriate developmental stage might represent a promising disease‐modifying strategy.^72^

#### Mitochondrial Inheritance

Disease phenotypes resulting from (predominantly) maternally inherited mutations in mtDNA are highly heterogeneous in terms of penetrance, expressivity and pleiotropy due to heteroplasmy, which is secondary to random segregation of mitochondria during cell replication (Fig 3G).^25^ Mature oocytes typically contain more than 100,000 mtDNA copies.^1^ If they carry a variant in at least one copy of mtDNA, any postzygotic cell can be homoplasmic for the wild‐type (ie, all mtDNA copies are wild‐type), homoplasmic for the mutant (ie, all mtDNA copies carry the mutation), or heteroplasmic (ie, wild‐type and mutant mtDNA molecules are present). Every child of an affected heteroplasmic mother inherits at least some mutant mtDNA copies whose proportion is difficult to predict. Moreover, the ratio of mutant to wild‐type mtDNA copies can change over time and between tissues, and the threshold for mutant mtDNA molecules to express into the phenotype is highly tissue specific. As a result, mutations in mtDNA can have low penetrance, extremely variable expressivity and pleiotropy, with rather unpredictable effects on the phenotype. Different levels of heteroplasmy can explain intrafamilial divergent phenotypes in mitochondrial DNA disorder.^26^ Low‐level and/or tissue‐limited heteroplasmy may represent major challenges for clinicogenetic diagnoses. NGS with deep sequence coverage and extraction of DNA from tissues of interest enhance sensitivity and allow for accurate quantification of heteroplasmy.^13^

### Epigenetic Factors

Penetrance and expressivity of genetic variants also depend on the activity status of loci carrying them. Epigenetic modifications are changes in gene expression which do not entail a change in DNA sequence and, although being not permanent, can be mitotically and/or meiotically heritable.^73^ These include DNA methylation, histone modification, and non‐coding RNA‐associated gene silencing.^73^ Epigenetic factors are responsible of genomic imprinting and X‐chromosome inactivation, and are also recognized in disorders whose primary molecular underpinning is either a genetic or chromosomal defect, as in FXS, where the “full” mutation in *FMR1* triggers epigenetic events that reduce/abolish its transcription.

#### Genomic Imprinting

Some AD disorders exhibit parent‐of‐origin effects; namely, the mutant allele can be transmitted by either parent, the disease phenotype is usually expressed only when the genetic variant is inherited from the mother or father.^1^ The best characterized phenomenon leading to parent‐of‐origin effect is genomic imprinting, that is the silencing of an allele through DNA methylation depending on the parent of origin (Fig 3H).^1^ For instance, the gene encoding ε‐sarcoglycan (*SGCE*) and accounting for ~1/3 of myoclonus‐dystonia is maternally imprinted.^17^, ^18^ Heterozygous variants in *SGCE* most often exhibit incomplete penetrance if they are inherited from the mother. Differential methylation of CpG dinucleotides in the promoter region of *SGCE* has been proven as molecular mechanism.^17^, ^18^ Individuals who carry the mutation in the imprinted allele usually do not manifest symptoms but can transmit it to the offspring, which results in apparent generation skipping. Intriguingly, in ~6% of individuals with *SGCE*‐related myoclonus‐dystonia, the pathogenic variant is transmitted by the mother.^16^ The reasons for loss of the maternal imprinting are unknown, but removal of differentially methylated regions or mutations in regions that are critical for imprinting regulation may be hypothesized. In these cases, the phenotype may be milder, thus contributing to intrafamilial variable expressivity.

#### X‐Chromosome Inactivation

X‐chromosome inactivation is the transcriptional silencing of one X chromosome in female mammalian cells in order to equalize X‐linked gene dosage between females and males.^1^ X‐inactivation is usually a random phenomenon, and females carrying a genetic variant on chromosome X are in fact mosaic, with each cell population functionally hemizygous.^1^ Hence, female carriers of genetic variants on chromosome X would be expected to produce approximately half of an abnormal gene product, thus manifesting milder phenotypes compared to males carrying the same variant, as often observed in FXTAS, FXS, and X‐linked dystonia‐parkinsonism (OMIM# 314250).^74^, ^75^ However, since X‐inactivation occurs in the early stages of female embryogenesis, there is wide variable expressivity of X‐linked mutant alleles in females. In addition, some X‐linked disorders may have a deleterious effect on cell function during early embryogenesis, a phenomenon that may lead to extreme skewing of the distribution of cell populations (Fig 3I). Finally, some genes escape X‐inactivation and are expressed from both the active and inactive X chromosome. Such genes are potential contributors to sexually dimorphic traits, to phenotypic variability among females heterozygous for X‐linked conditions, and to clinical abnormalities in patients with abnormal X chromosomes. All these mechanisms contribute to phenotypic heterogeneity in female carriers of a mutant allele on chromosome X.^1^

### Environmental Factors

The phenotype of some single‐gene disorders is also influenced by environmental factors, including the direct exposure of cells to harmful or potentially harmful chemicals (eg, dietary components), ionizing radiation, and traumas.

The first mechanism is exemplified by inborn errors of metabolism, which are due to deficiency of a single enzyme catalyzing a step in a specific metabolic pathway and manifest with the introduction of the substrate whose metabolism is defective. A paradigmatic example is phenylketonuria (OMIM# 261600), which is caused by biallelic mutations in the gene *PAH* encoding the enzyme responsible to convert phenylalanine into tyrosine. Defective enzymatic activity leads to severe intellectual disability in the context of a normal diet, whereas an early and relatively strict phenylalanine‐restricted diet results in a healthy phenotype or in mild neurological impairment.^76^

Another example of gene–environment interaction which can influence phenotypic heterogeneity is provided by ataxia‐telangiectasia (AT; OMIM# 208900). AT is a rare autosomal recessive disorder due to mutations in the *ATM* gene. *ATM* encodes a critical regulator of the cellular response to DNA double strand breaks. Individuals carrying mutations show hypersensitivity to ionizing radiation and a high incidence of cancer, primarily of lymphoid origin.^77^

Finally, in the context of DYT‐*TOR1A*, a positive association between a history of complications of vaginal delivery and manifestation of dystonia was demonstrated, thus suggesting that perinatal adversities might modulate penetrance in DYT‐*TOR1A*.^15^

### Unknown Factors

A significant proportion of incomplete penetrance, variable expressivity and pleiotropy is not explained by the aforementioned mechanisms. *ATP1A3‐* and *TUBB4*‐related disorders represent emblematic examples. *ATP1A3* encodes a subunit of the transmembrane Na+/K+ ATPase, which is the major contributor to rapid restoration of neuronal membrane potential after rapid depolarization. Monoallelic variants in *ATP1A3* have been associated with a number of neurological disorders, including alternating hemiplegia of childhood (AHC) type 2 (OMIM# 614820), rapid‐onset dystonia‐parkinsonism (RDP; OMIM# 128235), and the cerebellar ataxia, areflexia, *pes cavus*, optic atrophy and sensorineural hearing loss (CAPOS) syndrome (OMIM# 601338).^32^ Moreover, EKG dynamic abnormalities have been detected in all *ATP1A3*‐related syndromes, with a risk of life‐threatening cardiac rhythm abnormalities equivalent to that observed in cardiac channelopathies.^78^ Very recently, a distinct neonatal‐onset phenotype named D‐DEMØ has been linked to *ATP1A3*, encompassing dystonia, facial dysmorphism, encephalopathy with developmental delay, brain MRI abnormalities always associated with cerebellar hypoplasia, but absence of hemiplegia (Ø).^79^ Mechanisms underlying this wide phenotypic heterogeneity are controversial. Review of published cases revealed that the same genetic variant may account for different phenotypes (eg, RDP in one family, but AHC in another) and that there is a growing number of patients with intermediate and non‐classic phenotypes.

Monoallelic variants in *TUBB4A* account for hypomyelination with atrophy of the basal ganglia and cerebellum (H‐ABC) syndrome (OMIM# 612438), a rare neurodegenerative disorder of infancy and childhood, and for “hereditary whispering dysphonia” (formerly DYT4; OMIM# 128101), in which brain imaging is usually unremarkable.^33^ Cases with clinical phenotype characterized by severe generalized dystonia associated with pyramidal, bulbar and cerebellar features, but imaging findings at least partially consistent with H‐ABC syndrome have been reported, so that genotype–phenotype correlation is controversial.^34^

## Discussion

MD have emerged as paradigmatic example of challenging genotype–phenotype correlations due to their high degree of genotypic and phenotypic heterogeneity. This landscape is set to get even more intriguing with the advent of long‐read sequencing (single molecule real‐time sequencing and nanopore sequencing) and other technologies (eg, electronic nano‐device sequencing, nanochannel genome mapping) which promise to overcome intrinsic limitations of PCR‐based NGS.^60^ As proof of the ongoing new revolution in sequencing technology, over few years, a SINE‐VNTR‐Alu (SVA) retrotransposon insertion in *TAF1* has been suggested as the possible molecular cause of X‐linked dystonia‐parkinsonism, with a hexameric repeat expansion within the SVA insertion acting as a genetic modifier of disease expressivity.^75^, ^80^ Moreover, intronic pentanucleotide repeat expansions were detected in different genes related to familial cortical myoclonic tremor syndromes,^81^ and a trinucleotide repeat expansion in *NOTCH2NLC* was linked to neuronal intranuclear inclusion disease.^82^ Most of these findings also shed light to the relatively unexplored non‐coding regions of the genome in the etiopathogenesis of MD and neurodegenerative diseases.

Some considerations arise from our overview of the phenomenological and mechanistic bases of phenotypic heterogeneity in monogenic disorders, which ultimately fuels the complexity of clinicogenetic correlations.

First, establishing that a specific genetic variant in a particular individual with a given disorder is the molecular underpinning responsible of its phenotype does not allow to automatically infer that this mutant genotype will invariably lead to the same phenotype in all individuals harboring it. This can only be established (or refuted) empirically by comprehensive, ideally prospective, analysis of the genotype in question.

Second, determining to what extent genetic test results correlate with clinical characteristics will be an increasingly demanding challenge in the post‐NGS era. Indeed, WES and WGS detect in each individual several thousand and a few million sequence variants that differ from the human genome reference, respectively. The evaluation of variant pathogenicity (ie, the probabilistic assertion of the likelihood that the variant is disease‐causing) and the integration of genetic findings with the phenotypic features and family history of an affected individual are steps of a complex, multidisciplinary process to establish a genetic diagnosis, involving bioinformaticians, geneticists and clinicians. Always more often MD specialists will be required to formulate challenging pathogenicity assertions by matching data collected in the clinical arena with findings from genetic testing.

Understanding the relationship between genotype and phenotype is the cornerstone of precision medicine and a must for clinicians in the post‐NGS era. Automated processes and machine learning algorithms have hitherto failed to provide accurate genotype‐to‐phenotype prediction, which limits their use in clinical diagnostics and neurogenetic research. Despite their expected refinement in the next future, it is likely that they will never replace the role of clinicians and geneticists entirely, especially in the MD field, which relies on fine‐grained clinical judgment to define phenotypes without parallel in neurology. For all these reasons, the exponential growth of genetic knowledge driven by NGS has reaffirmed the central role of meticulous clinical phenotyping. More specifically, it has promoted individual‐oriented “deep phenotyping”,^83^ ie, the detailed and comprehensive analysis of discrete components of a phenotype that goes beyond what is typically recorded in clinical charts (eg, nuanced phenotypic traits, such as “short stature” or “mild dysmorphic features”), generally in a way which is computationally accessible and enables to integrate the resulting wealth of data with non‐clinical information. In other words, a transition from the “definition” to the “holistic characterization” of phenotypes has been started since the NGS era. In clinical practice, deep phenotyping will further guide clinicians through differential diagnosis, selection of genetic tests and interpretation of their results, targeted therapeutic interventions and genetic counseling. In the research setting, it will help to expand the knowledge on established genotype–phenotype correlations or in determining novel ones.

In conclusion, deep phenotyping, with characterization and continual updating of “core” phenotypes, and comprehension of determinants of genotype–phenotype complex relationships are crucial for clinicogenetic correlations and will have always more implications for diagnosis, treatment and genetic counseling.

## Author Roles

(1) Research project: A. Conception, B. Organization, C. Execution; (2) Data Analysis: A. Design, B. Execution, C. Review and Critique; (3) Manuscript Preparation: A. Writing of the first draft, B. Review and Critique.

F.M.: 1B, 1C, 3A

B.B.: 3B

K.P.B.: 1A, 1B, 3B

## Disclosures

### Ethical Compliance Statement

We confirm that we have read the Journal's position on issues involved in ethical publication and affirm that this work is consistent with those guidelines. The authors confirm that the approval of an institutional review board was not required for this work. The authors confirm that no patient consent was required for this work.

### Funding Sources and Conflict of Interest

No specific funding was received for this work. The authors declare that there are no conflicts of interest relevant to this work.

### Financial Disclosures for the Previous 12 Months

FM is supported by the European Academy of Neurology (EAN) Research Fellowship 2020. BB reports no disclosures. KPB has received grant support from Welcome/MRC, NIHR, Parkinson's UK and EU Horizon 2020. He receives royalties from publication of the Oxford Specialist Handbook Parkinson's Disease and Other Movement Disorders (Oxford University Press, 2008), of Marsden's Book of Movement Disorders (Oxford University Press, 2012), and of Case Studies in Movement Disorders–Common and uncommon presentations (Cambridge University Press, 2017). He has received honoraria/personal compensation for participating as consultant/scientific board member from Ipsen, Allergan, Merz and honoraria for speaking at meetings and from Allergan, Ipsen, Merz, Sun Pharma, Teva, UCB Pharmaceuticals and from the American Academy of Neurology and the International Parkinson's Disease and Movement Disorders Society.
